# First-Trimester Prenatal Dexamethasone Treatment Is Associated With Alterations in Brain Structure at Adult Age

**DOI:** 10.1210/clinem/dgaa340

**Published:** 2020-06-04

**Authors:** Annelies van’t Westeinde, Leif Karlsson, Anna Nordenström, Nelly Padilla, Svetlana Lajic

**Affiliations:** Department of Women’s and Children’s Health, Karolinska Institutet, Pediatric Endocrinology Unit, Karolinska University Hospital, Stockholm, Sweden; Department of Women’s and Children’s Health, Karolinska Institutet, Pediatric Endocrinology Unit, Karolinska University Hospital, Stockholm, Sweden; Department of Women’s and Children’s Health, Karolinska Institutet, Pediatric Endocrinology Unit, Karolinska University Hospital, Stockholm, Sweden; Department of Women’s and Children’s Health, Karolinska Institutet, Division of Neonatology, Karolinska University Hospital, Stockholm, Sweden; Department of Women’s and Children’s Health, Karolinska Institutet, Pediatric Endocrinology Unit, Karolinska University Hospital, Stockholm, Sweden

**Keywords:** dexamethasone, prenatal treatment, brain structure, epigenetics, mood, cognition

## Abstract

**Context:**

Prenatal treatment of human disease is rare. Dexamethasone (DEX) is used in pregnancies at risk for congenital adrenal hyperplasia (CAH) to prevent virilization in an affected female fetus. The safety and long-term consequences of prenatal DEX exposure on the brain are largely unknown.

**Objective:**

We investigate whether first-trimester prenatal DEX treatment is associated with alterations in brain structure at adult age, and if these alterations are associated with DNA methylation, mood, and cognitive abilities.

**Design, Setting, and Participants:**

T1-weighted and diffusion-weighted imaging scans, from a single research institute, are compared between 19 (9 women) first-trimester DEX-treated individuals, at risk of CAH but not having CAH, and 43 (26 women) controls (age range, 16.0-26.4 years).

**Results:**

DEX-treated participants showed bilateral enlargement of the amygdala, increased surface area and volume of the left superior frontal gyrus, and widespread increased radial, mean, and axial diffusivity of white matter, in particular in the superior longitudinal fasciculi and corticospinal tracts. In the DEX-treated group, increased mean and radial diffusivity correlated with increased methylation of the promotor region of the *FKBP5* gene. There were no group differences in cognition or in scales assessing depression or anxiety, and the relationship between brain structure and cognition did not differ between DEX-treated and controls.

**Conclusions:**

First-trimester prenatal DEX treatment is associated with structural alterations of the brain at adult age, with an accompanying change in gene methylation. The findings add to the safety concerns of prenatal DEX treatment in the context of CAH.

Congenital adrenal hyperplasia (CAH) is mainly caused by mutations in the *CYP21A2* gene, resulting in impaired production of cortisol and aldosterone in the adrenal cortex (1). CAH ranges in severity from the most severe salt-wasting form (SW), to the milder simple-virilizing (SV), and finally nonclassic CAH. In SW CAH, the aldosterone deficiency may lead to early salt-losing crisis and neonatal death. SV CAH on the other hand is diagnosed during childhood due to accelerated growth and precocious pseudopuberty, whereas nonclassic CAH is often detected during adolescence or even in adult age because of irregular menstrual periods, hirsutism, and infertility problems (2, 3). Neonatal screening programs are used in many countries worldwide and detect nowadays most cases of classic CAH (SW and SV). Hormone replacement therapy with glucocorticoid (GC) and mineralocorticoid may thus be initiated in the second week of life (4).

A female fetus with classic CAH will become virilized already in utero because of overproduction of adrenal androgens. In addition to genital virilization, affected females might display more male-typical behavior (5). To prevent prenatal virilization, the synthetic GC dexamethasone (DEX) has been given to women who previously gave birth to a child with classic CAH (6). Recent clinical guidelines however no longer recommend the practice outside a clinical trial (7). To be effective, treatment must be initiated before gestational week (GW) 7, that is, before differentiation of the external genitalia, and continue to term in case of a female fetus with CAH . This practice results in unnecessary treatment in 7 out of 8 pregnancies, because CAH is an autosomal recessive disorder and prenatal genotyping is usually not possible prior to GWs 10 to 12. In boys and healthy girls, treatment is stopped after the first trimester but the dose the fetus is exposed to is 60 times the physiological level of cortisol during that time period of gestation (8, 9). The long-term consequences of the excess prenatal GC exposure are largely unknown, but might lead to sequelae or negative effects on cognition later in life (10). A follow-up study in Sweden found impairments in verbal working memory in DEX-treated children without CAH between ages 7 and 17 years (11), with more pronounced effects in girls, who also had reduced verbal and nonverbal intelligence compared to healthy nontreated controls (12). However, at adult age these differences in working memory seemed to attenuate, even though the participants did not improve in verbal intelligence over time (13). The neural mechanisms underlying the long-term effects of prenatal DEX treatment on cognition are thus far unknown. Exposure to DEX from GWs 7 to 12 coincides with a sensitive period of neurogenesis and neuronal migration (14). In addition, epigenetic mechanisms might cause long-term programming effects on brain development. We previously found altered methylation status in genes relevant for brain development and function in DEX-treated individuals not having CAH, including brain-derived neurotrophic factor (*BDNF*), the GC receptor (GR) *NR3C1*, the mineralocorticoid receptor *NR3C2*, and the GR co-chaperone *FKBP5* gene (15). These genes are involved in hypothalamic-pituitary-adrenal (HPA) axis function, and altered methylation of some of these genes has been linked with prenatal and early life stress (16-18), depression (19, 20), as well as changes in structure of the brain during adolescence, as is the case for *BDNF* (18). Prenatal programming effects of excess synthetic GCs might therefore lead to altered brain structure and HPA axis sensitivity. This could have consequences for cognitive function and mood regulation that depend largely on networks with a high density of GRs (21).

Research in humans has previously mostly focused on second- and third-trimester effects of cortisol exposure on child neurodevelopment. Synthetic GC exposure during the third trimester, given to induce fetal lung maturation in cases at risk of preterm birth, was associated with reduced thickness of the rostral anterior cingulate cortex (22). Higher prenatal maternal cortisol levels, even within the normal physiological range, are associated with reduced fetal brain growth (23), altered gray-matter volumes (24, 25) and altered functional (26) and structural connectivity (27) during childhood. The amygdala, which develops very early in fetal life, seems to be especially vulnerable to early disturbances in cortisol levels (28, 29), and increased amygdala volume is associated with increased symptoms of depression in girls (24, 27). However, no human studies have so far investigated the effects of first-trimester GC exposure on brain development.

Understanding the impact of prenatal DEX treatment in the context of CAH is crucial to assess and determine treatment safety and perform risk-benefit analyses. Because the treatment holds no benefit for most individuals, no negative side effects can be tolerated. Based on the early development of the amygdala and findings from previous studies, we hypothesize that the amygdala might be particularly vulnerable to early cortisol disturbances. The present study aimed to investigate: 1) whether first-trimester treatment with DEX is associated with structural brain alterations of gray and white matter at adult age, specifically amygdalae volumes, 2) whether structural brain alterations correlate with changes in DNA methylation, and 3) whether these changes predispose for problems with cognitive performance and increased mood symptoms. We studied a group of adolescent and adult prenatally DEX-exposed participants at risk of CAH but not having CAH and healthy population controls.

## Methods

### Participants

The participants were selected from the PREDEX study (investigating the effects of prenatal and postnatal GC treatment in the context of CAH) (12, 13, 15, 30) and included only participants not having CAH who had received prenatal DEX treatment during the first trimester (range start DEX, GWs 5-9; range stop DEX, GWs 10-22; mean duration DEX, 6 weeks [range, 1.5–14 weeks]) and nontreated control individuals from the general population (13). The control participants were unrelated to the DEX-treated participants and all lived in the Stockholm County area. They were recruited from the Swedish Civil Registration System based on the age and sex distribution of the DEX cohort. The present analyses were based on n = 19 (9 female) DEX-treated participants, mean age = 20.7 years (2.6); age range, 16.3 to 26.4 years; and n = 43 (26 female) controls, mean age = 20.3 years (2.7); age range, 16.1 to 25.3 years. The study was approved by the Regional Ethics Committee of Stockholm (No. 99-153 and 1658-32), and all participants and parents of children younger than 18 years gave their informed consent before study inclusion.

### Procedure

The study procedure has been described previously in detail (13, 15, 30). Briefly, the participants completed neuropsychological tests and, on a separate day (mean difference 263 days, range 0-800 days), a 70-minute magnetic resonance imaging (MRI) brain scan on a 3T MR scanner (Discovery MR750, General Electric) with an 8-channel head coil. Structural T1-weighted images, T2 weighted images, and diffusion-weighted imaging (DWI) acquisitions were used in the study. Self-reported well-being was obtained after the scanning according to a 10-point visual analog scale. Participants were asked to report any health-related problems in a screening questionnaire regarding health and lifestyle. No health problems were reported by the participants.

Neuropsychological tests included verbal and nonverbal intelligence (Wechsler Adult Intelligence Scale [WAIS]-IV Vocabulary and WAIS-IV Matrices [31], executive functions and working memory performance (WAIS-IV Digit Span [31] and Span Board Test [32]), learning and memory (Wechsler Memory Scale [WMS]-III List Learning Test [32]), processing speed and interference (WMS-III Coding [32] and the Stroop Task [33]). Self-rated questionnaires were used for assessment of executive functioning (Barkley Deficit in Executive Functioning Scale–Short Form [34]) and depressive and anxiety symptoms (Hospital Anxiety and Depression Scale [35, 36] and Liebowitz Social Anxiety Scale–Self-Report [37]).

### Data acquisition and analyses

Estimates of subcortical volumes, and vertex-wise cortical surface area, volume, and thickness (FreeSurfer) and gray-matter volumes (voxel-based morphometry, VBM) were obtained from T1-weighted images (brain volume imaging sequence, repetition time [TR] = 7.9 ms, echo time [TE] = 3.1 ms, 176 slices, voxel size: 1.0 × 1.0 × 1.0 mm). Estimates of white-matter integrity were obtained using FSL-TBSS (Tract-Based Spatial Statistics [38]) based on diffusion-weighted scans (TR = 7.4 seconds, 62 slices, voxel size: 2.3 × 2.3 × 2.3 mm, 60 DWI (b = 1500 s/mm^2^) and 8 images with no diffusion sensitization [b = 0 s/mm^2^]).

#### Analysis of cortical thickness, surface area and cortical and subcortical volumes.

The default FreeSurfer 6 pipeline was implemented (http://surfer.nmr.mgh.harvard.edu/) (39, 40). The constructed surfaces were checked and manually corrected by 2 independent researchers blinded to the participant’s condition, until satisfied about the quality. Estimates of subcortical volumes of 9 × 2 hemispheres = 18 regions were obtained from automatic segmentation. Our main focus was on assessing bilateral amygdala volumes. In addition, exploratory analyses were performed on volumes of the subcortical regions other than the amygdala (n = 16 regions). False discovery rate (FDR) correction was applied on the exploratory subcortical analyses (n = 16), and q values less than 0.05 were considered significant.

In addition, we used the FreeSurfer Qdec application to investigate cortical thickness, surface area and volume in a whole-brain vertex-wise analysis, and applied Monte Carlo simulation using prerun data in Qdec with a significance level of *P* less than .05 (threshold 1.3) using a 2-sided test and n = 10 000 permutations.

#### Analyses of structural data for voxel-based morphometry.

Anatomical T1 images for all participants were analyzed with an optimized VBM protocol (41) implemented in FSL-VBM (42), http://fsl.fmrib.ox.ac.uk/fsl/fslwiki/FSLVBM; part of the FSL tools, (43). Briefly, T1 images were brain-extracted using the brain extraction tool, segmented to obtain subject specific gray-matter maps (FAST), registered into standard space (MNI 152) using nonlinear registration in FNIRT (44, 45) and used to create a study-specific gray-matter template. To prevent biases toward one of the groups in the template, equal numbers of participants per group were included in the template creation, by random selection of individuals from the control group to match the number of the DEX-treated group. Thus, the template is based on n = 38 participants. Native gray-matter images were registered to the study-specific template using FNIRT, “modulated” to correct for local expansion or contraction, and smoothed with a 3-mm isotropic Gaussian kernel. Voxel-wise statistics were carried out using FSL’s randomize tool. Clusters were defined using threshold free cluster enhancement (46, 47), with 10 000 permutations and localized using the Harvard-Oxford Cortical Structural Atlas.

#### Analysis of diffusion tensor imaging data.

We estimated fractional anisotropy (FA), mean diffusivity (MD), axial diffusivity (AD), and radial diffusivity (RD) using FSL’s TBSS tool. Eddy correction was applied to the DWI acquisitions to correct for eddy currents and motion, using the new eddy-correct paradigm without top-up, but with the -repol option to replace outliers related to motion (48). Eddy-corrected data were brain-extracted and FA images were created by fitting a tensor model to the raw diffusion data using FDT (49). The FA data were then aligned into a common space using the nonlinear registration tool FNIRT (50). The mean FA image was created and thinned to create a mean FA skeleton, which represents the centers of all tracts common to the group. Each participant’s aligned FA data were then projected onto the skeleton and the resulting data were fed into voxel-wise cross-individual statistics. The nonlinear warps and skeleton projection were also applied to participants’ MD, AD, and RD images using the tbss_non_FA script provided by TBSS tools.

One nontreated control participant (female) did not have good-quality diffusion tensor imaging images and was excluded from the analysis. Voxel-wise statistical analysis of diffusion tensor imaging data was carried out using FSL’s randomize tool. For each measure, clusters were defined using threshold free cluster enhancement, with 10 000 permutations per contrast. Significant clusters were localized using the JHU White-Matter Tractography Atlas.

### Statistical analysis

#### Effects of prenatal dexamethasone treatment on gray- and white-matter brain structure.

Group comparisons between DEX-treated and controls were made for age and general well-being using one-way analysis of variance. The effect of DEX treatment on whole-brain volume, subcortical volume (FreeSurfer), neocortical volume, surface area and thickness (FreeSurfer QDEC), gray-matter volume (FSL-VBM), and white-matter microstructure (FSL-TBSS) was assessed in 3 steps: 1) DEX-treated vs controls, 2) interaction between DEX treatment and sex, and 3) post hoc analyses splitting males and females, that is, DEX-treated women vs control women and DEX-treated men vs control men, for those tests showing a significant interaction term after multiple comparisons correction. All analyses in step 1 were corrected for age, sex, and total brain volume, and all analyses in step 2 and 3 were corrected for age and total brain volume. An estimate of total brain volume was creating using BrainSegVolNotVent (brain volume without ventricles) software for FreeSurfer, and summed gray- and white-matter volume from FSL-FAST for FSL-VBM and FSL-TBSS. Multiple comparisons corrections were performed based on the appropriate method per software, as described above, for steps 1 and 2. Step 3 is considered post hoc in nature; therefore, no correction was applied. Based on the outcome of these analyses, we further investigated whether the structure of the identified regions that differed between DEX-treated individuals and controls was associated with degree of gene methylation, cognitive performance, and mood symptoms in DEX-treated participants.

#### Associations between gene methylation and gray- and white-matter structure in dexamethasone-treated participants.

We tested the association between degree of methylation of CpG sites of genes that were previously found to be differentially methylated in non-CAH DEX-treated individuals, that is *FKBP5*, *BDNF*, *NR3C1, NR3C2*, and *SLC6A4* (15) and brain structure estimates that differed between DEX-treated cases and controls: amygdala, left superior frontal cortex, and white-matter microstructure. The analysis was performed on a subset of the data for which methylation information was available, n = 14 DEX-treated (5 female), mean age 21.25 years (range, 16.32-26.39 years), and n = 16 nontreated controls (10 female), mean age 21.60 (range, 18.46-25.32 years). DNA methylation data were acquired from our previous study (15), in which we analyzed CD4+ T cell DNA using the Infinium Human Methylation 450K BeadChip array (15). A linear regression model was employed with methylation degree predicting brain structure, correcting for demeaned values of age, sex, and total brain volume. The analyses were conducted in 3 steps: 1) whole-group associations, 2) interactions between DEX and methylation, and 3) post hoc tests splitting DEX-treated and controls for those associations that showed a significant interaction term after multiple comparisons correction. FDR correction was performed for the number of methylation sites (n = 14) in steps 1 and 2, q and *P* values less than .05 were considered significant. This approach was chosen because we were interested to see whether the relationship between gene methylation and brain structure differed significantly between DEX-treated and untreated controls. Owing to the small sample size, we were not able to assess sex effects for this step.

#### Associations between cognitive skills and mood symptoms and brain structure in dexamethasone-treated participants.

Between-group differences in cognitive performance and internalizing symptoms, that is, anxiety and depression, were analyzed using analyses of variance, again using 3 steps: 1) whole-group analyses, 2) interaction between DEX and sex, and 3) post hoc tests splitting men and women for those tests that showed a significant interaction term after multiple comparisons correction. FDR correction was applied for the number of cognitive and behavioral tests (n = 16) in steps 1 and 2. Next, we tested the association between brain structure and cognitive performance and behavioral tests in the whole group using linear models. In addition, we ran interaction analyses to test whether there was a group difference between DEX-treated and controls for the association between the cognitive and behavioral test and the brain regions that we observed to be different between groups. FDR correction was applied for the number of cognitive and behavioral scales.

#### Total number of comparisons.

In addition to the whole-brain voxel and vertex-wise analyses, we performed 335 comparisons (TBV [n = 1], bilateral amygdala [n = 2], bilateral subcortical regions of interest [ROIs] [n = 16], methylation associated with white matter [n = 56; 14 sites × 4 estimates], methylation associated with gray matter [n = 84; 14 sites × 6 ROIs], cognition and behavior associated with white matter [n = 64; 16 scales × 4 estimates], cognition and behavior associated with gray matter [n = 96; 16 scales × 6 ROIs], and group comparisons on cognition and behavior [n = 16 scales]). At a *P* value of .05 for 335 comparisons, we would expect approximately 17 false positives, which is why we conducted FDR correction. In addition, interaction analyses were performed on these tests, giving a total of 670 comparisons.

Several associations between behavior and brain, and between methylation and brain, were found that did not survive FDR correction. Raw results from the subcortical analyses and linear associations between gene methylation and ROIs and between cognitive and behavior scales and ROIs can be found in the supplementary information published online (51). These provide the uncorrected *P* values for the interested reader.

## Results

### Demographics

There were no group differences in age and overall well-being between DEX-treated participants (mean age, 20.3 years) and control participants at the time of scanning (mean age, 20.7 years) (Table 1).

**Table 1.** Demographic characteristics of the study population. Data are shown as mean (SD)

	Female group		Male group		
	DEX (f)	C (f)	DEX (m)	C (m)	F statistics DEX vs C
No.	9	26	10	17	
Age, y	20.44 (2.54)	19.96 (2.49)	20.91(2.82)	20.78 (2.99)	F (1, 60) = 0.30, *P* = .58
Well-being^*a*^	7.22 (1.29)	7.49 (0.89)	7.31 (1.57)	7.41 (1.47)	F (1, 58) = 3.12, *P* = .083

Abbreviations: C, control; DEX, dexamethasone; f, female; m, male.

^*a*^ General well-being, continuous 10-point visual analog scale.

#### Occurrence of structural abnormalities.

Structural abnormalities, as observed by an independent radiologist as part of the routine protocol at the MR center, from T2-weighted images were found in one DEX-treated individuals (5.3%): a corpus pineal cyst, and in 8 controls (18.6%): 2 participants had Arnold-Chiari malformation type 1, 4 had minor WM changes, and 2 had a Rathke cyst. The difference between 1 observation in DEX and 8 in controls was not significant (chi-square with Yates correction = 0.968, *P* = .325).

#### Effects of prenatal dexamethasone treatment on whole-brain and subcortical volumes.

There was no group difference in total brain volume. DEX-treated participants had increased volume of the bilateral amygdala compared to controls (left 8.31% larger [F (1, 57) = 6.79, *P* = .012, Cohen’s *d *= 0.65]; right 9.06% larger [F (1, 57) = 7.53, *P* = .008, Cohen’s *d *= 0.74]), but there were no interactions with sex. Explorative analyses of the other subcortical regions (n = 16, 8 bilateral) showed no significant group differences or interactions between DEX and sex (Supplementary Table 1) (51).

#### Effects of prenatal dexamethasone treatment on neocortical cortical volume, surface area, and thickness (Qdec).

**Figure 1.**
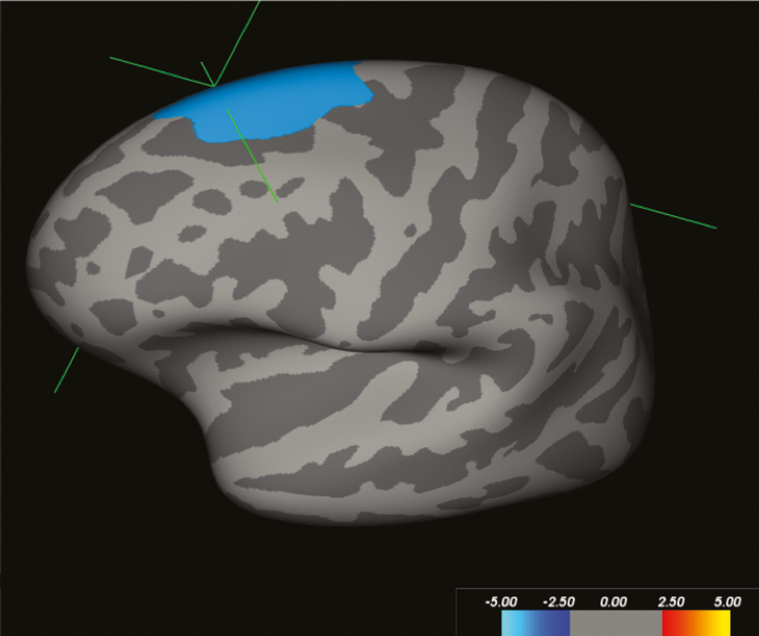
FreeSurfer Qdec results for dexamethasone (DEX)-treated vs control participants, corrected for sex, age, and brain volume, showing increased surface area of the left superior frontal gyrus (size 2320.77 mm^2^, coordinates TalX = –22.7, TalY = 24.0, TalZ = 51.0, Max = –4.00, *P* < .001). Monte Carlo–simulated data on n = 62, degrees of freedom = 54, 10 000 permutations, threshold 1.3 (*P* < .05).

DEX-treated participants had increased surface area (size 2320.77 mm^2^, coordinates TalX = –6.6, TalY = 33.8, TalZ = 49.8, max = –4.00, *P* < .001) (Fig. 1) and volume (size 1837.98 mm^2^, coordinates TalX = –6.6, TalY = 33.8, TalZ = 49.8, max = –4.00, *P* < .001) of the left superior frontal gyrus. There were no interactions with sex.

#### Effects of prenatal dexamethasone treatment on gray-matter volumes using voxel-based morphometry.

There was no difference in gray-matter volume between DEX-treated and controls and there were no interactions with sex.

#### Effects of prenatal dexamethasone treatment on white-matter microstructure.

**Figure 2.**
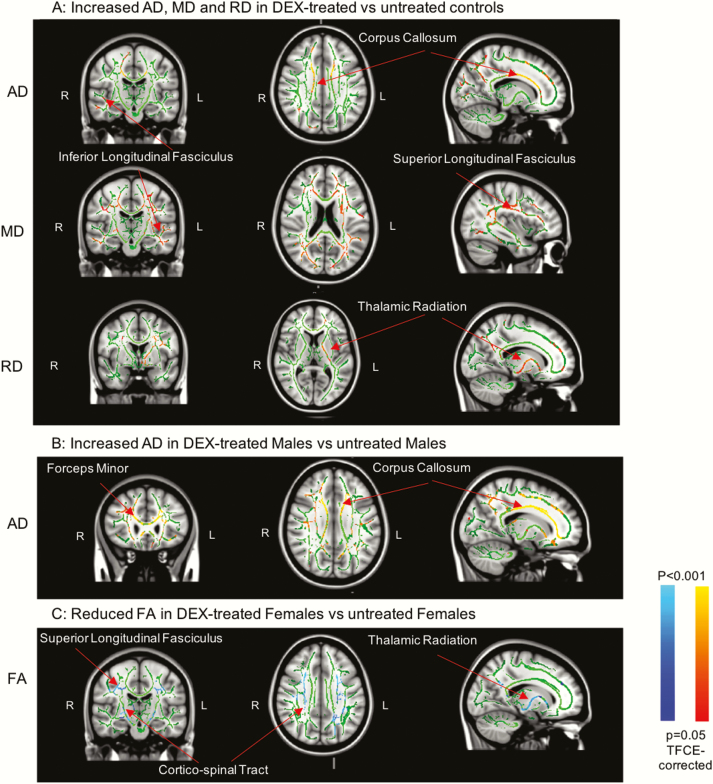
White-matter microstructure alterations in dexamethasone (DEX)-treated vs control participants (threshold free cluster enhancement [TFCE]-corrected with 10 000 permutations). A, Increased AD in the corpus callosum and right inferior longitudinal fasciculus, increased MD in the left inferior and superior longitudinal fasciculi, and increased RD in the left thalamic radiation. B, increased AD in DEX-treated men, in particular in the forceps minor and corpus callosum. C, reduced FA in DEX-treated women, in particular in the right corticospinal tract, right superior longitudinal fasciculus, and left anterior thalamic radiation. *Orange and red colors indicate increased mean values of AD, MD, and RD in the DEX-treated group compared to controls. Blue colors indicate reduced mean values of FA in the DEX-treated female group compared to nontreated control women. Lighter colors (light-blue and yellow) indicate a lower *P* value compared to darker colors (dark blue and red), with the highest *P* value of .05 after TFCE correction being represented by the darkest shades of red and blue. AD, axial diffusivity; FA, fractional anisotropy; MD, medial diffusivity; RD, radial diffusivity. Green = mean FA skeleton across groups. Examples of tracts that differed between groups are indicated by red arrows.

DEX-treated participants had widespread increases in RD (left anterior thalamic radiation, left corticospinal tract, superior longitudinal fasciculus, and right cingulum), MD (bilateral superior longitudinal fasciculus, bilateral corticospinal tract, and corpus callosum), and AD (right inferior fronto-occipital fasciculus, left anterior thalamic radiation, and right inferior and superior longitudinal fasciculus) compared to controls (Fig. 2A). In addition, there were interactions between DEX-treatment and sex for FA and AD. Post hoc comparisons revealed that DEX-treated men had increased AD (forceps minor and major, bilateral inferior fronto-occipital fasciculus, bilateral superior longitudinal fasciculus, right corticospinal tract, and corpus callosum), whereas DEX-treated women had reduced FA (bilateral thalamic radiation, bilateral corticospinal tract, and bilateral superior longitudinal fasciculus (Fig. 2B and 2C).

#### Association between gene methylation and gray-matter structure.

There were no associations between gene methylation and gray-matter structure in the combined group of DEX-treated and controls, and there were no interactions between DEX treatment and gene methylation (Supplementary Table 2A and 2C) (51).

#### Association between gene methylation and white-matter microstructure.

There were no associations between gene methylation and white-matter microstructure in the entire study group. However, there was a significant interaction between DEX treatment and gene methylation for methylation of the *FKBP5* promotor region (cg00130530, 5’UTR;TSS1500) on mean RD (B = 0.0004, q = 0.035). Post hoc comparisons revealed that increased methylation of the *FKBP5* promotor region (cg00130530) was associated with increased mean RD (B = 0.0004, *P* = .003) only in the DEX-treated group (Supplementary Table 2B and 2C) (51).

#### Effects of prenatal dexamethasone treatment on cognitive skills and mood symptoms.

Mean scores on cognitive tasks and self-rated mood symptoms for the DEX-treated participants and controls are found in Tables 2 and 3 respectively. There were no group differences on cognitive performance between DEX-treated and controls, and there were no interactions with sex. Moreover, brain structure did not predict cognitive performance or behavior in the whole group, and there were no interactions between DEX treatment and brain structure on any of the scales (Supplementary Table 3A-3C) (51).

**Table 2.** Cognitive measures for dexamethasone-treated participants and controls. Mean values (SD) are presented. There were no significant group differences

	DEX (n = 19)			Control (n = 43)		
	Total	Female (n = 9)	Male (n = 10)	Total	Female (n = 26)	Male (n = 17)
General IQ						
WAIS-IV Matrices	12 (3.28)	11.22 (2.44)	12.70 (3.89)	11.63 (3.05	10.73 (2.62)	13.00 (3.22)
WAIS-IV Vocabulary	9.95 (1.58)	9.33 (1.50)	10.50 (1.51)	9.88 (2.78)	9.50 (3.08)	10.47 (2.21)
Executive functions						
WAIS-IV Digit span	10.05 (2.50)	9.78 (3.07)	10.30 (2.00)	10.30 (2.83)	9.92 (3.07)	10.88 (2.39)
WAIS-IV Coding	11.26 (2.49)	11.56 (1.74)	11.00 (3.09)	10.47 (2.89)	11.31 (2.43)	9.18 (3.13)
WMS-III Span board (forward)	10.16 (3.56)	9.22 (3.53)	11.00 (3.56)	10.74 (2.91)	10.62 (2.58)	10.94 (3.44)
WMS-III Span board (backward)	11.68 (1.83)	11.11 (1.90)	12.20 (1.69)	12.28 (1.75)	12.50 (1.70)	11.94 (1.82)
Stroop interference test	54.79 (14.37)	51.89 (20.03)	57.40 (6.36)	54.65 (10.59)	52.69 (12.52)	57.65 (5.81)
B-DEFS-SF	32.32 (8.10)	30.11 (6.60)	34.30 (9.12)	32.77 (9.03)	34.08 (7.43)	30.76 (11.00)
Learning and memory						
WMS-III Wordlist (LTM)	11.84 (2.54)	11.67 (2.06)	12.00 (3.02)	12.23 (2.80)	12.27 (3.29)	12.18 (1.91)
WMS-III Wordlist (STM)	11.05 (2.61)	10.89 (2.71)	11.20 (2.66)	10.44 (2.90)	10.62 (3.13)	10.18 (2.58)

Abbreviations: B-DEFS-SF; Barkley Deficit in Executive Functioning Scale–Short Form; DEX, dexamethasone; LTM, long-term memory; STM, short-term memory; WAIS, Wechsler Adult Intelligence Scale; WMS, Wechsler Memory Scale.

**Table 3.** Behavioral measures for dexamethasone (DEX)-treated participants and controls. Mean values (SD) are displayed. There were no significant group differences between DEX-treated cases and controls

	DEX (n = 19)			Control (n = 43)		
	Total	Female (n = 9)	Male (n = 10)	Total	Female (n = 26)	Male (n = 17)
Mood						
HADS Anxiety	5.79 (3.24)	7.00 (3.32)	4.70 (2.91)	6.52 (3.46)	7.40 (3.88)	5.18 (2.19)
HADS Depression	3.16 (2.14)	2.67 (1.66)	3.60 (2.50)	3.16 (2.83)	2.77 (2.52)	3.76 (3.23)
LSAS Anxiety	13.63 (9.93)	13.44 (8.37)	13.80 (11.61)	16.05 (10.81)	16.12 (12.33)	15.94 (8.32)
LSAS Avoidance	12.89 (10.09)	13.11 (8.42)	12.70 (11.85)	16.23 (10.69)	15.73 (11.41)	17.00 (9.77)
LSAS Total	26.53 (19.21)	26.56 (16.08)	26.50 (22.54)	32.28 (20.58)	31.85 (22.88)	32.94 (17.14)
AQ10	1.79 (1.62)	1.67 (1.94)	1.90 (1.37)	1.84 (1.43)	1.85 (1.29)	1.82 (1.67)

Abbreviations: AQ, Autism Quotient; HADS, Hospital Anxiety and Depression Scale; LSAS, Liebowitz Social Anxiety Scale.

## Discussion

Here we show that first trimester (GWs 7-12) prenatal DEX treatment in individuals at risk for CAH, but not having CAH, results in brain structure alterations at adult age. We observed increased amygdala volume, increased volume and surface area of the left superior frontal gyrus, and widespread alterations in white-matter microstructure, namely increased AD, RD, and MD in particular in the superior longitudinal fasciculi and corticospinal tracts. In addition, DNA methylation of the *FKBP5* gene in CD4+ T cells was associated with altered white-matter microstructure (RD) in DEX-treated cases. However, we observed no group differences in cognitive performance or symptoms of anxiety and depression. Thus, although prenatal DEX treatment was associated with substantial brain structure alterations, we could not detect problems with cognition or behavior in this adult cohort.

Our findings support the fetal programming effects of early prenatal GC treatment on development of the brain. The organizational effect of elevated prenatal cortisol levels on brain development has previously been shown for second- (24) and third- (22, 23, 27) trimester maternal cortisol levels (26), but not before GW 15. Naturally varying prenatal cortisol levels have been shown to be associated with changes both in infant and childhood brain structure, including altered regional gray-matter volumes, amygdala connectivity, and neural network properties (23-27). Third-trimester treatment with synthetic GCs (betamethasone) has been linked with cortical thinning in childhood (22). Buss and colleagues already suggested that elevated cortisol levels earlier in pregnancy, as opposed to later, might have a more pronounced effect on childhood brain development (24). Our findings indicate that already in very early embryonic stages, when neurogenesis is ongoing and neurons start migrating to their locations in the cortex (14), heightened GC levels alter the developmental trajectory of the brain.

These alterations might occur through a direct impact of GCs on the developing neurons, by affecting expression of genes in neural stem/progenitor cells (52-55) or by epigenetic programming of genes involved in brain development and function, in particular the HPA axis. We observed bilateral enlargement of the amygdala in DEX-treated participants in accordance with previous studies (24, 27). The amygdala is likely to be vulnerable to early cortisol disturbances because it is one of the first structures to develop, around GW 8.5 (28, 29) and expresses GC receptors in high density (56, 57). Interestingly, reduced amygdala volumes have been found in patients with CAH who are treated with GC-replacement medication postnatally, and who have thus experienced prenatal lack of cortisol (58). Thus, prenatal and postnatal imbalances or prenatal lack or excess in cortisol might have reversed effects on amygdala development. In contrast to the amygdala, the superior frontal cortex and axonal myelination develop largely postnatally (59, 60). It might therefore be expected that, for these structures in particular, epigenetic mechanisms underlie the observed alterations in DEX-treated individuals. Indeed, heightened prenatal GC exposure results in altered gene transcription in the prefrontal cortex across several generations (61). However, notably there was no group difference in hippocampal volume, despite this structure being known to contain a high density of cortisol receptors and to be sensitive to disturbances in cortisol levels (55, 62).

Further, oligodendrocytes and their precursors express GC receptors (63), and postnatal oligodendrocyte differentiation and myelination require the presence of cortisol (64). Thus, disturbances in HPA axis development resulting in altered cortisol levels later in life (65), or epigenetic mechanisms resulting in differential GR expression in precursor cells, might affect white-matter development. In a previous study we observed differential methylation of genes that are relevant for brain development and glucocorticoid signaling and action in healthy DEX-treated participants (15). Here we show that white-matter microstructure in adulthood is associated with increased methylation of a CpG site in the gene body of a promotor region of the gene for the GR co-chaperone *FKBP5*. The *FKBP5* gene is involved in HPA-axis function and might therefore mediate the effect of prenatal GCs on postnatal HPA-axis components, such as cortisol levels (17). Altered methylation of *FKBP5* has previously been associated with depression (19, 20) and stress-dependent gene transcription (66). *FKBP5* therefore poses a potent mechanism through which first-trimester DEX treatment could exert its long-term effects on the brain, either as a direct effect, or as part of a compensatory mechanism ameliorating the initial effect.

The observed structural alterations did not seem to be associated with accompanying changes in cognition or mood. However, analyses from the same cohort at a younger age (age 7-17 years) did reveal significant problems with cognition, which were more pronounced in girls (12), but which seemed to have attenuated by adult age (13). Indeed, neither in our previous study at adult age (13), nor in the present study on the same participants (excluding those who did not undergo MR scanning or were excluded from the MR analyses: n = 4 DEX-treated and n = 15 controls), were there differences in cognition or mood in the DEX-treated group. It is possible that the effect size of a potential group difference has become too small in adults to detect with our sample size. More sensitive measures, both in terms of cognitive functioning as well as in terms of MRI analyses, might be necessary to detect an association between cognition and brain structure. Further, it must be noted that the mean anxiety scores in the present study (m = 26.56 in women, m = 26.50 in men) were substantially lower in the DEX-treated women compared to the scores from the same cohort in a previous report (m = 35.3 in women, m = 25.7 in men) (13). It appears that the DEX-treated women with high anxiety had simply not undergone the MRI scanning procedure. It is therefore difficult to make definitive conclusions about the relationship between the observed brain structure changes and mood symptoms in the DEX-treated population. In addition, the present study used self-reported estimates of mood symptoms. A more careful examination based on expert interviews might be needed to rule out clinically relevant symptoms of anxiety and depression to which the observed neuroendophenotype might predispose.

One possible explanation for the absence of an association between brain structure and cognition or behavior could be that the organization of the brain in the DEX-treated individuals has adapted to the circumstances, potentially using compensatory mechanisms, to catch up (67). One could hypothesize that the observed structural alterations might thus have contributed to the normalization of brain function into adulthood. Alternatively, despite a difference in brain structure or genetic makeup at birth, the association between function and structure might develop in a healthy way. However, without a longitudinal design we are not able to draw any conclusions regarding these hypotheses. Moreover, the group differences in structure might be associated with differences in functional activation of the brain that account for the compensatory mechanisms in terms of cognition and behavior.

Despite the lack of cognitive or behavioral changes, the fact that we observe a large change in amygdala size, increased size of the superior prefrontal gyrus, and widespread alterations in white-matter microstructure that are associated with epigenetic changes does add to the concern about the safety of first-trimester DEX treatment in the context of CAH. Indeed, recent practice guidelines from the Endocrine Society have already been adapted and prenatal DEX treatment is no longer recommended as standard of care in pregnancies at risk for CAH, but should be obtained only in research settings or centers large enough for proper assessment of risks and benefits (7). Our findings support this conclusion and point toward a need for adapting the treatment protocol to completely avoid treatment in fetuses that do not benefit from DEX treatment.

### Strengths and limitations

The strengths of this study include a well-defined and rare cohort, characterized prenatally and followed prospectively. A possible limitation is the relatively small sample size, which may have prevented statistical differences from being observed in some comparisons. Indeed, subthreshold findings, that is, associations with *P* less than .05 that did not survive FDR correction, were observed for several brain behavior and brain-methylation estimates, as are reported in the supplementary materials online (51). In addition, group sizes were unequal, with one substantially larger control group. Though this should not affect the outcome per se, it might have reduced our power to detect significant effects. Moreover, brain maturation continues well into the mid-20s and differences in brain structure between DEX-treated individuals and controls might therefore not be the same for those in their late teens compared to mid-20s. This difference is not captured in our analyses, even when using age as a covariate. Studies with a larger sample including smaller age bins would be needed to test whether the difference in brain structure changes in the age range of our sample. Finally, we would like to stress that we used T-cell methylation as a proxy for methylation in general and for methylation in brain cells in this case. Although a correlation between methylation of blood and brain regions has been shown (68), we are aware that methylation changes may be tissue specific and even variable within brain regions. In addition, methylation of different gene regions of the *FKBP5* gene might have different effects on GR function (69). Moreover, the effect of altered immune response peripherally to GCs on the brain cannot be disregarded either. Hence, we refrain from speculating about the exact mechanisms through which *FKBP5* methylation might affect mood in DEX-treated individuals.

Nevertheless, the combination of different MRI modalities and approaches provide an excellent opportunity to elucidate the underlying structural substrates of early treatment in the context of CAH.

## Conclusions

Taken together, our findings show that first-trimester DEX exposure results in altered brain structure at adult age, which is associated with epigenetic changes in a promotor region of the *FKBP5* gene. Despite structural changes, we could not detect significant differences in cognition or mood in the DEX-treated participant compared to controls in this particular cohort. However, subthreshold associations and a lack of participation of participants with higher anxiety strongly suggest that research on more DEX-treated individuals is needed to exclude an effect of prenatal treatment on cognition and behavior at adult age. The findings do contribute to concerns about the safety of prenatal DEX treatment in the context of CAH, and of prenatal GC treatment in general. The fact that we see changes in brain structure that persists into adult age, and changes in methylation, known to be of epigenetic nature, further strengthens the concern and points toward a decision that early prenatal treatment should not be used in fetuses that do not benefit from the treatment per se. The findings have implications for the clinical management of CAH and warrant the development of methods for earlier prenatal diagnosis in both sexes to avoid unnecessary treatment in healthy babies.

## Abbreviations

AD: axial diffusivity
BDNF: brain-derived neurotrophic factor
CAH: congenital adrenal hyperplasia
DEX: dexamethasone
FA: fractional anisotropy
FDR: false discovery rate
GC: glucocorticoid
GR: glucocorticoid receptor
GW: gestational week
HPA: hypothalamic-pituitary-adrenal
MD: mean diffusivity
MRI: magnetic resonance imaging
RD: radial diffusivity
ROI: region of interest
SV: simple-virilizing
SW: salt-wasting
VBM: voxel-based morphometry
WAIS: Wechsler Adult Intelligence Scale
WMS: Wechsler Memory Scale

## Additional Information

***Disclosure Summary:*** The authors have nothing to disclose.

***Data Availability:*** Restrictions apply to the availability of data generated or analyzed during this study to preserve patient confidentiality or because they were used under license. The corresponding author will on request detail the restrictions and any conditions under which access to some data may be provided.
